# The mature phyllosphere microbiome of grapevine is associated with resistance against *Plasmopara viticola*

**DOI:** 10.3389/fmicb.2023.1149307

**Published:** 2023-04-11

**Authors:** Wisnu Adi Wicaksono, Christina Morauf, Henry Müller, Ahmed Abdelfattah, Christina Donat, Gabriele Berg

**Affiliations:** ^1^Institute of Environmental Biotechnology, Graz University of Technology, Graz, Austria; ^2^SAN Agrow Holding GmbH, Herzogenburg, Austria; ^3^Leibniz Institute for Agricultural Engineering and Bioeconomy (ATB), Potsdam, Germany; ^4^Bio-Ferm GmbH, Getzersdorf, Austria; ^5^Institute for Biochemistry and Biology, University of Potsdam, Potsdam, Germany; ^6^Austrian Centre of Industrial Biotechnology (ACIB GmbH), Graz, Austria

**Keywords:** phyllosphere, microbiome, grapevine, *Plasmopara viticola*, amplicon 16S rRNA

## Abstract

Phyllosphere microbiota represents a substantial but hardly explored reservoir for disease resistance mechanisms. The goal of our study was to understand the link between grapevine cultivars susceptibility to *Plasmopara viticola,* one of the most devastating leaf pathogens in viticulture, and the phyllosphere microbiota. Therefore, we analyzed a 16S rRNA gene library for the dominant phyllosphere bacterial phyla *Alphaproteobacteria* of seven *Vitis* genotypes at different developmental stages, i.e., flowering and harvesting, *via* amplicon sequencing. Young leaves had significantly higher Alphaproteobacterial richness and diversity without significant host-specificity. In contrast, the microbial communities of mature leaves were structurally distinct in accordance with *P. viticola* resistance levels. This statistically significant link between mature bacterial phyllosphere communities and resistant phenotypes was corroborated by beta diversity metrics and network analysis. Beyond direct host-driven effects *via* the provision of microhabitats, we found evidence that plants recruit for specific bacterial taxa that were likely playing a fundamental role in mediating microbe-microbe interactions and structuring clusters within mature communities. Our results on grape-microbiota interaction provide insights for targeted biocontrol and breeding strategies.

## Introduction

Plant surfaces are complex micro-ecosystems referred to as the phyllosphere which harbors a large fraction of commensal or mutualistic bacteria, archaea, fungi, and protists that positively affect the health and growth of their hosts (Vorholt, 2012; Liu et al., 2020). Fluctuations in UV radiation and temperature as well as water and nutrients heterogeneous result in an ephemeral, and unstable biotope for phyllosphere inhabitants (Vorholt, 2012). Apart from environmental variables and geospatial dispersion patterns of microorganisms (Bokulich et al., 2014; Wicaksono et al., 2023), factors related to phenotypic traits such as leaf morphology (Hunter et al., 2010; Kusstatscher et al., 2020), cuticle synthesis (Bodenhausen et al., 2014; Ritpitakphong et al., 2016) or volatile and hormone signaling (Vorholt, 2012) account for the selection of a distinct microbiome. Moreover, previous investigations have shown a strong impact of urban intensity (Laforest-Lapointe et al., 2017), crop management practices (Grube et al., 2011; Knorr et al., 2019; Chen et al., 2021), and season (Redford and Fierer, 2009; Rastogi et al., 2012) on the phyllosphere microbiome. Previous work indicated site-specific, consistently re-occurring patterns between years (Redford and Fierer, 2009; Knief et al., 2010; Müller et al., 2016). However, the phyllosphere is a less explored reservoir for extended host functions by microbes such as disease resistance (Zhan et al., 2022).

The genus *Vitis* comprises approximately 60 species with diversity centers in North America, Eastern Asia, and Europe (Alleweldt et al., 1991; Wan et al., 2013). Nevertheless, most of the cultivars grown on 7.2 million hectares worldwide and produced 79 million tons of grapes are classified as *Vitis vinifera* subsp. *vinifera,* which is susceptible to downy mildew, one of the most destructive grapevine diseases (Jürges et al., 2009). *Vitis* species endemic in North America such as *V. labrusca, V. riparia, and V. rupestris* are more resistant to downy mildew, most likely because of their longer co-evolution with the causative agent *Plasmopara viticola* (Berk. and Curt.) Berl. and de Toni (Alleweldt and Possingham, 1988; Schröder et al., 2011).

Resistance against *P. viticola* includes constitutive mechanisms detracting stomatal infection by zoospores such as trichomes and reducing the wettability of the abaxial side of the leaves (Kortekamp et al., 1999) or the presence of an inner cuticular rim at the neck region of the substomatal cavity (Jürges et al., 2009). Induced resistance, on the other side, depends on the recognition of unspecific pathogen-associated molecular patterns (PAMPs) and of specific effector proteins secreted particularly by host-adapted biotrophs to evade PAMP-triggered immunity. Successful pathogen recognition as described by Jones and Dangl (Jones and Dangl, 2006) is the result of a co-evolutionary process between host and pathogen leading to the accumulation of pathogenesis-related proteins, reactive oxygen species, and phenolic compounds within the grapevine plant (Gindro et al., 2006; Malacarne et al., 2011; Toffolatti et al., 2012).

So far, however, the grapevine phyllosphere microbiota has not been considered for its role in conferring resistance. Studies on *Arabidopsis* (Horton et al., 2014; Ritpitakphong et al., 2016), maize (Balint-Kurti et al., 2010; Manching et al., 2014; Wallace et al., 2018; Wagner et al., 2020), and olive (Hladnik et al., 2022) suggest a relationship between microbial communities in the phyllosphere and susceptibility to leaf pathogens. There is already evidence that bacterial communities in the phyllosphere are selected by host genotype effects like microstructural leaf traits and molecular signaling (Hunter et al., 2010; Bodenhausen et al., 2014; Wagner et al., 2016; Wallace et al., 2018; Kusstatscher et al., 2020). However, knowledge of how host plant genetic variation and their specific microbial communities are interfering with susceptibility to leaf pathogens is scarce. Breeding for broad-spectrum disease resistance in maize lead to an alteration in the colonization success of leaf pathogens but also affected the not pathogenic microbial phyllosphere community (Wallace et al., 2018, 2020). Therefore, we hypothesize that the phyllosphere microbiota of grape is correlated with the resistance toward *P. viticola*. To evaluate our hypothesis, we studied the *Vitaceae* collection grown in the Botanical Garden in Graz (Austria). This unique facility provides equal climate and soil conditions without chemical plant protection. Starting with a first sampling operation in October examining a mature microbial leaf community, we consequently conducted a follow-up experiment in June to consider the well-known impact of the plant development stage on associated microbiota, particularly in the composition and dynamics of *Alphaproteobacteria*, a major phylogenetic group that dominates the plant phyllosphere (Vorholt, 2012; Laforest-Lapointe et al., 2016). This group of taxa is also known to contribute to the health and productivity of the plant host, i.e., N_2_ fixation, phosphate solubilization, and protection against plant pathogens (Madhaiyan et al., 2015; Alibrandi et al., 2018; Matsumoto et al., 2021; Wicaksono et al., 2021).

## Materials and methods

### Plant material and sampling procedure

Leaves were collected from the Botanical Garden in Graz (Austria) where 34 taxa of the family *Vitaceae*—true species as well as hybrids—grow in close vicinity to each other allowing for avoidance of inhomogeneous climatic and soil-driven influences. Samples of the chosen grapevine cultivars (Table 1) were randomly taken in four replicates and compared to susceptible “Müller Thurgau,” plants kept under controlled conditions in a greenhouse in Tulln (Austria). As only green and asymptomatic leaves from the shoot tips of healthy plants were investigated in our study, we excluded leaves from susceptible “Blauer Wildbacher” plants in October as they were clearly influenced by pathogen infection. In neither of the sample sites, classical viticultural measures like canopy management, tillage, or fungicide treatments were performed. Sampling took place in October 2014 and June 2015. According to the BBCH-scale for grapes (Lorenz et al., 1995), the phenological plant development stages were 89 (berries ripe for harvest) in October and 69 (end of flowering) in June.

**Table 1:** Characteristics of grapevine species and cultivars investigated in this study.

*Vitis* genotype	Primary origin	*P. viticola* resistance level	Stomatal rim	Growing site	References
*Vitis amurensis* Rupr.	Northeast Asia (Amur region)	Highly resistant	Yes	Botanical Garden Graz	Staudt and Kassemeyer (1995), Boso and Kassemeyer (2008)
*Vitis vinifera* L. subsp. *sylvestris* (C.C. Gmelin) Beger	Southeast Europe	Moderately resistant	No	Botancial Garden Graz	Schröder (2010)
*Vitis vinifera* L. subsp. *vinifera* “Blauer Wildbacher”	Europe (Austria)	Susceptible	No	Botanical Garden Graz	Teppner (2003)
*Vitis labrusca* L.	Eastern North America	Lowly resistant	No	Botanical Garden Graz	Cadle-Davidson (2008)
*Vitis* x *alexanderi* Prince ex Jacques “Isabella”		Lowly resistant	No	Botanical Garden Graz	Cadle-Davidson (2008)
*Vitis riparia* L.	Central and Eastern North America	Highly resistant	Yes	Botanical Garden Graz	Staudt and Kassemeyer (1995), Boso and Kassemeyer (2008)
*Vitis vinifera* L. subsp. *vinifera* “Müller Thurgau”	Europe (Germany, Switzerland)	Susceptible	No	Greenhouse Tulln	Staudt and Kassemeyer (1995), Boso and Kassemeyer (2008)

Per sample, a defined leave area of 20 × 18 cm was treated for 3 min and two times 1 min with 50 mL sterile 0.85% sodium chloride solution containing 0.01% Tween 80 in a lab blender (BagMixer; Interscience, St. Nom, France) in alteration with 3 min sonication at 60 Hz. After centrifugation (16,000 × g, 20 min, 4°C) and removal of the supernatant, the microbial pellet was harvested and stored at −70°C for further processing.

### Confocal laser scanning microscopy using fluorescence *in **situ*** hybridization

To visualize natural colonization patterns of phyllosphere inhabiting bacteria, fluorescence *in situ* hybridization in combination with Confocal Laser Scanning Microscopy (CLSM) was conducted according to Cardinale et al. (2008). Small sections of paraformaldehyde-fixed leaves were incubated with lysozyme for 10 min and exposed to an ethanolic series (50–70-96% EtOH solutions; 3 min each). Two hybridization steps combining *Rhizobiales*-specific RHIZ3r probe and an equimolar mix of EUB338 probes targeting all bacteria were performed according to their stringency conditions (Erlacher et al., 2015). NONEUB was used as a negative control with formamide concentrations and fluorochrome labeling analog to the positive FISH probes. Not overlapping emission spectra of the fluorochromes allowing for differentiated signal detection were observed with a Leica TCS SPE confocal laser-scanning microscope (Leica Microsystems, Mannheim, Germany) containing solid-state and UV lasers. An additional channel was used for acquiring the autofluorescence of the leaf cells excited with a 405 nm laser beam and an emission range from 415 to 465 nm. Confocal stacks were attained with a Leica ACS APO 40X OIL CS objective (NA: 1.15) using a Z-step of 0.8 μm and averaging three scans per optical slice.

### DNA extraction, amplification, and Illumina sequencing

Microbial DNA obtained from grapevine leaves was extracted according to the manufacturer’s instructions using FastDNA Spin Kit for Soil (MP Biomedicals, Solon, OH, United States) and a FastPrep Instrument (BIO101 Systems, Qbiogene, Carlsbad, CA, United States) facilitating cell homogenization. The concentration of extracted DNA was spectrophotometrically determined (NanoDrop 2000c; Thermo Scientific, Wilmington, MA, United States). DNA templates were, in the first step of a nested PCR approach, amplified using the *Alphaproteobacteria-*specific primers ALF28f (5’-ARC GAA CGC TGG CGG CA-3′) (Ashelford et al., 2002) and ALF986r (5′-GGT AAG GTT CTG CGC GTT-3′) (Amann et al., 1997). Ten microliter of PCR mixture contained 5 x Taq-&GO (MP Biomedicals, Illkirch, France), 25 mM MgCl_2_, 10 μM of both primers, and 1 μL of DNA template (cycling conditions: 96°C, 4 min; 30 cycles of 96°C, 1 min; 54°C, 1 min; 74°C, 1 min; and final elongation at 74°C, 10 min). Consequently, the PCR product was diluted 1:100 and used for a second PCR targeting the V4 region of the 16S rRNA gene with the universal primer pair 515f (5’-GCC AGC MGC CGC GGT A-3′) and 806r (5’-ACT ACH VGG GTW TCT A-3′) (Caporaso et al., 2011) containing barcodes for multiplexing. Thirty microliter of PCR reaction mixture contained 5 × Taq-&GO (MP Biomedicals, Germany), 5 μM of both primers, and 1.2 μL of DNA template (cycling conditions: 94°C, 3 min; 32 cycles of 94°C, 45 s; 60°C, 1 min; 72°C, 18 s; and final elongation at 72°C, 10 min). After purification using the Wizard SV Gel and PCR Clean-Up system (Promega, Madison, WI, United States), the partial 16S rRNA gene library was sequenced *via* Illumina’s MiSeq platform (2 × 250 bp paired-end reads) by sequencing provider LGC Genomics (Berlin, Germany). The raw data were deposited at the European Nucleotide Archive (ENA) under project number PRJEB59055.

### Data analysis

The generated sequence data were processed by the quantitative insights into microbial ecology 2 (QIIME2) v.2022.2.0 (Bolyen et al., 2019). The reads were quality filtered, trimmed, denoised, and merged with the implemented DADA2 algorithm (Callahan et al., 2016) followed by the removal of chimeric sequences. The acquired amplicon sequence variants (ASVs) were taxonomically classified using the VSEARCH classifier (Rognes et al., 2016) based on the reference database Silva v128 (Pruesse et al., 2007). Before further analysis, all reads assigned to not *Alphaproteobacteria*, and mitochondria were removed. The final dataset contained 1,941,666 reads of alphaproteobacterial reads that were assigned to 2,109 ASVs.

To account for uneven sequencing depth, the datasets were normalized by rarefying to the lowest number of reads and using MetagenomeSeq’s cumulative sum scaling [CSS; (Paulson et al., 2013)] for subsequent alpha and beta diversity analysis, respectively. Differences in the alpha diversity based on the number of ASVs (species richness), Shannon index (bacterial diversity), and Faith’s phylogenetic diversity (PD Faith) were analyzed using the Kruskal–Wallis test. Beta diversity indices were estimated based on Bray–Curtis and weighted Unifrac distances (Lozupone and Knight, 2005) and subjected to Analysis of similarities (*ANOSIM*, 999 permutations) to test for significant effects of experimental factors on alphaproteobacterial community structures. Furthermore, edgeR (Robinson et al., 2010) was used to identify differentially abundant bacterial ASVs between sample groups. Bacterial ASVs were defined as significantly different if the *P_adjusted_* value was less than 0.1.

For the detection of ecologically relevant relationships between alphaproteobacterial taxa, we used Sparse Correlations for Compositional data (SparCC), which removes compositional effects and calculates correlation matrices for absolute abundances of ASVs (Friedman and Alm, 2012). For every pair of bacteria tested, 100 bootstraps of randomly selected bacteria with 20 iterations each were calculated before generating average positive and negative correlation coefficients and two-sided pseudo-*p* values. A co-occurrence network of ASVs with significant (*p* values <0.01) positive or negative correlations (coefficients ≥0.5 and ≤ −0.5) was plotted using Cytoscape 3.3.0 (Shannon et al., 2003). The network’s topological properties were calculated using Network Analyzer in Cytoscape (Assenov et al., 2008) with edges treated as undirected.

## Results

### Leaf morphological traits of investigated grapevines and their microbial colonization patterns

**Figure 1:**
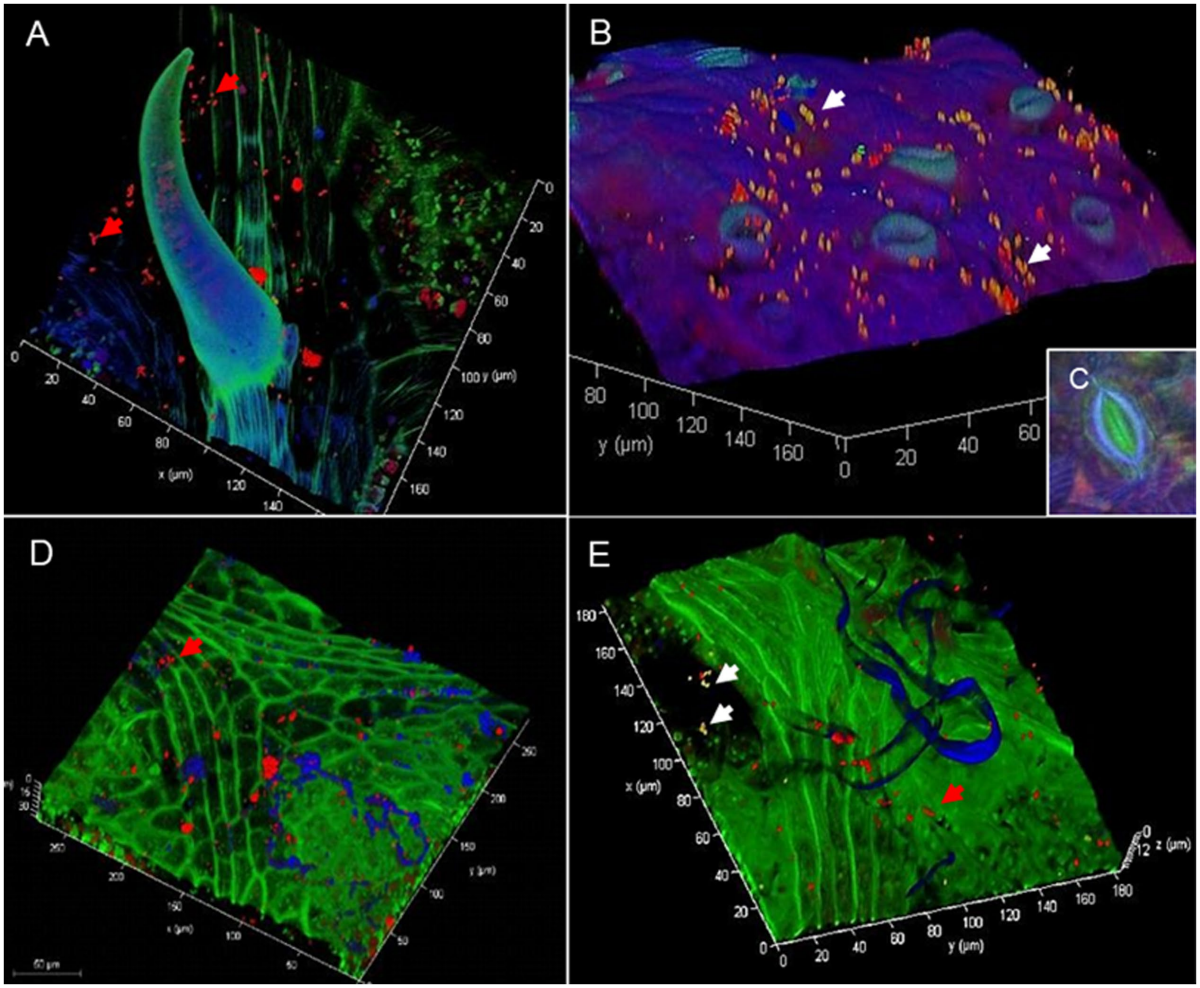
Confocal laser scanning microscopy (CLSM) micrograph in combination with fluorescence *in situ* hybridization showing bacterial colonization on grapevine phyllosphere. Confocal laser scanning micrographs of *Vitis riparia* leaves showing bacterial colonization patterns on nearby a trichome **(A)** and young, freshly emerged stomata **(B)** White arrows: *Rhizobiales*, red: other bacteria. *Vitis riparia* is considered to be highly resistant to *P. viticola* due to its inner stomatal rim **(C)**. Confocal laser scanning micrographs of *Vitis alexanderi* “Isabella” leaves showing bacterial colonization of leaf hairs **(D)** and nearby fungal hyphae and spores present on the leaf surface **(E)**. White arrows: *Rhizobiales*, red arrows: other bacteria, blue: autofluorescence of leaf hairs and fungal structures.

Colonization patterns in the *Vitis* phyllosphere were visualized by FISH/CLSM showing bacteria aggregated in colonies or individually along fungal hyphae (Figure 1), epidermal grooves, along leaf veins, and at the base of trichomes (Figure 1A). RHIZ3r-labeled bacteria indicating *Rhizobiales* were particularly observed on young leaves with freshly emerged stomata (Figure 1B). Leaf morphological characteristics such as the inner stomatal rim typical for *V. riparia* (Figure 1C) and *V. amurensis* were detected but did not display any obvious differences in bacterial colonization. *Vitis alexanderi* “Isabella”—an interspecific hybrid between *V. vinifera* and *V. labrusca*—are both characterized by a very high density of leaf hairs which were also partly colonized by *Rhizobiales* as well as by other bacteria (Figures 1D,E).

### Plant development was the major factor that affected alphaproteobacterial diversity and community structures

**Figure 2:**
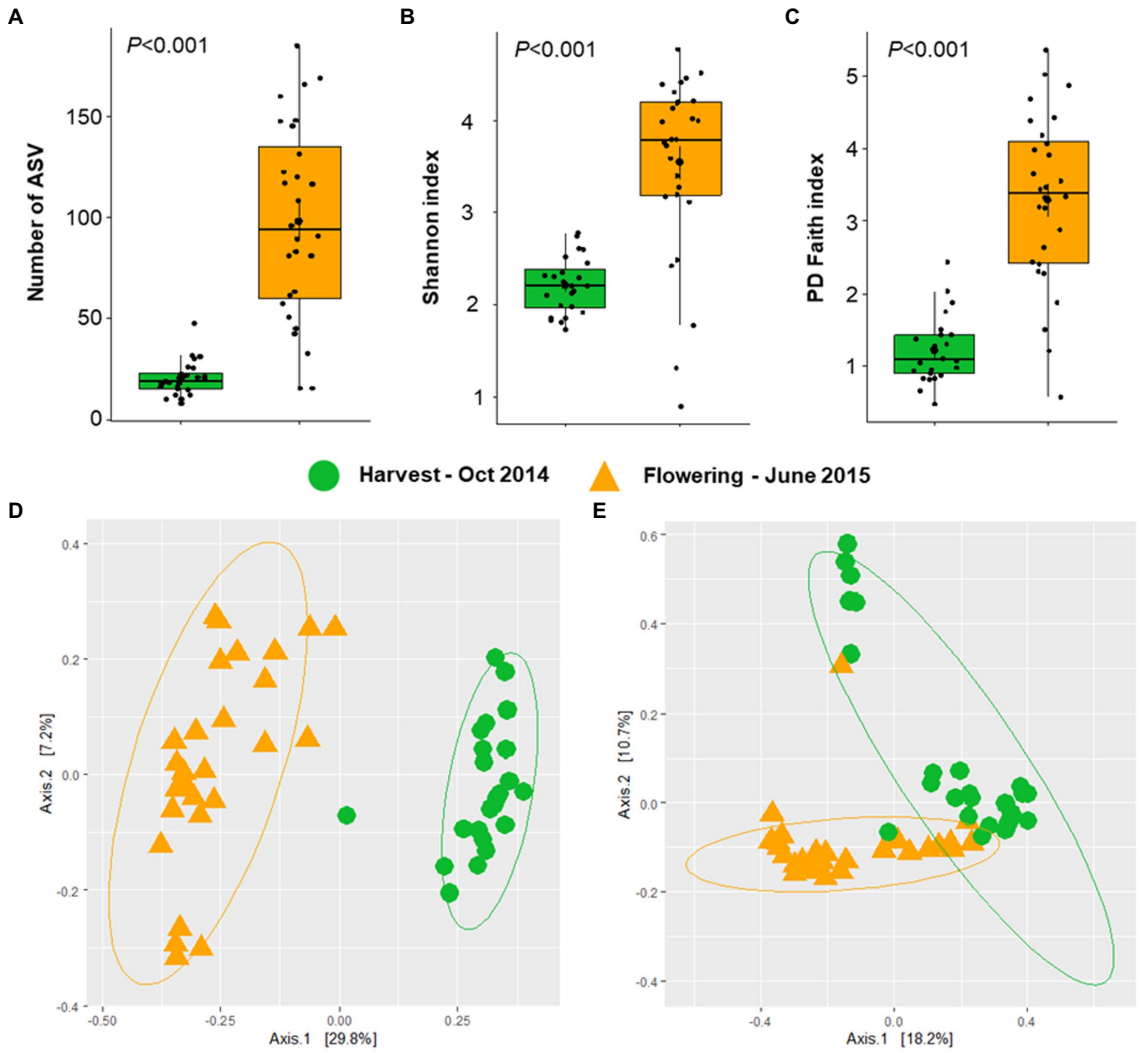
Alphaproteobacterial diversity and community structure at two phenological growth stages. Alphaproteobacterial richness and diversity were estimated based on the number of ASVs **(A)**, Shannon index **(B)**, and PD Faith index **(C)**. Significances in alphaproteobacterial diversity **(A–C)** were determined with the Kruskal Wallis test, representing *p* < 0.05. Alphaproteobacterial community clustering was assessed based on Bray–Curtis **(D)** and weighted UniFrac **(E)** matrix distances and visualized PCoA plots.

The alphaproteobacterial leaf microbiota of seven different *Vitis* accessions acquired at two phenological growth stages was examined in this study. Rarefaction curves based on the number of ASVs were saturated (Supplementary Figure S1). Indices for alphaproteobacterial richness (number of observed ASVs) and diversity (Shannon and PD faith index) were calculated. Interestingly, the species richness and diversity were significantly higher (Kruskal–Wallis test, *p* < 0.001) at the flowering period (number of observed ASV = 97.8; Shannon index = 3.5; PD faith index = 3.3, Figures 2A–C) than at harvesting period (number of observed ASV = 20.3; Shannon index = 2.2; PD faith index = 1.2).

Beta diversity analysis indicated that the plant developmental stage had a significant impact on the alphaproteobacterial community structure using abundance profiles collected from amplicon sequencing data (Bray–Curtis–ANOSIM, *R* = 0.770, *p* = 0.001; weighted UniFrac–ANOSIM, *R* = 0.562, *p* = 0.001). According to the PCoA plots that were generated using Bray–Curtis and weighted UniFrac distance matrices, two clusters were observed, confirming the significant differences in the alphaproteobacterial community structure according to the two plant developmental stages - flowering and harvesting (Figures 2D,E). The PCoA plot showed that the first two PCs explained 37.0 and 28.2% of the cumulative variances, respectively.

### Alphaproteobacterial community structures were explained by grapevine cultivar and resistance traits against *Plasmopara viticola*

To investigate cultivar effects on the alphaproteobacterial community, the data were separately analyzed according to the plant developmental stages. According to alpha diversity analysis, grapevine cultivar did not significant effect on the number of observed ASVs (Kruskal–Wallis test, *p* = 0.360, and *p* = 0.178), Shannon index (*p* = 0.202 and *p* = 0.712), and PD faith index (*p* = 0.434 and *p* = 0.253) at flowering and harvesting periods. Interestingly, at the flowering period, grapevine cultivars significantly affected alphaproteobacterial community structure (Bray–Curtis–ANOSIM, *R* = 0.286, *p* = 0.001; weighted UniFrac–ANOSIM, *R* = 0.271, p = 0.001). Differences in alphaproteobacterial community structure between different grapevine cultivars were smaller at the harvesting period (Bray–Curtis–ANOSIM, R = 0.132, *p* = 0.036; weighted UniFrac–ANOSIM, *R* = 0.191, *p* = 0.003) in comparison to the flowering period. Pairwise comparison indicated that cultivar “Müller Thurgau” had a different alphaproteobacterial community in comparison to other cultivars (*p* < 0.05).

**Figure 3:**
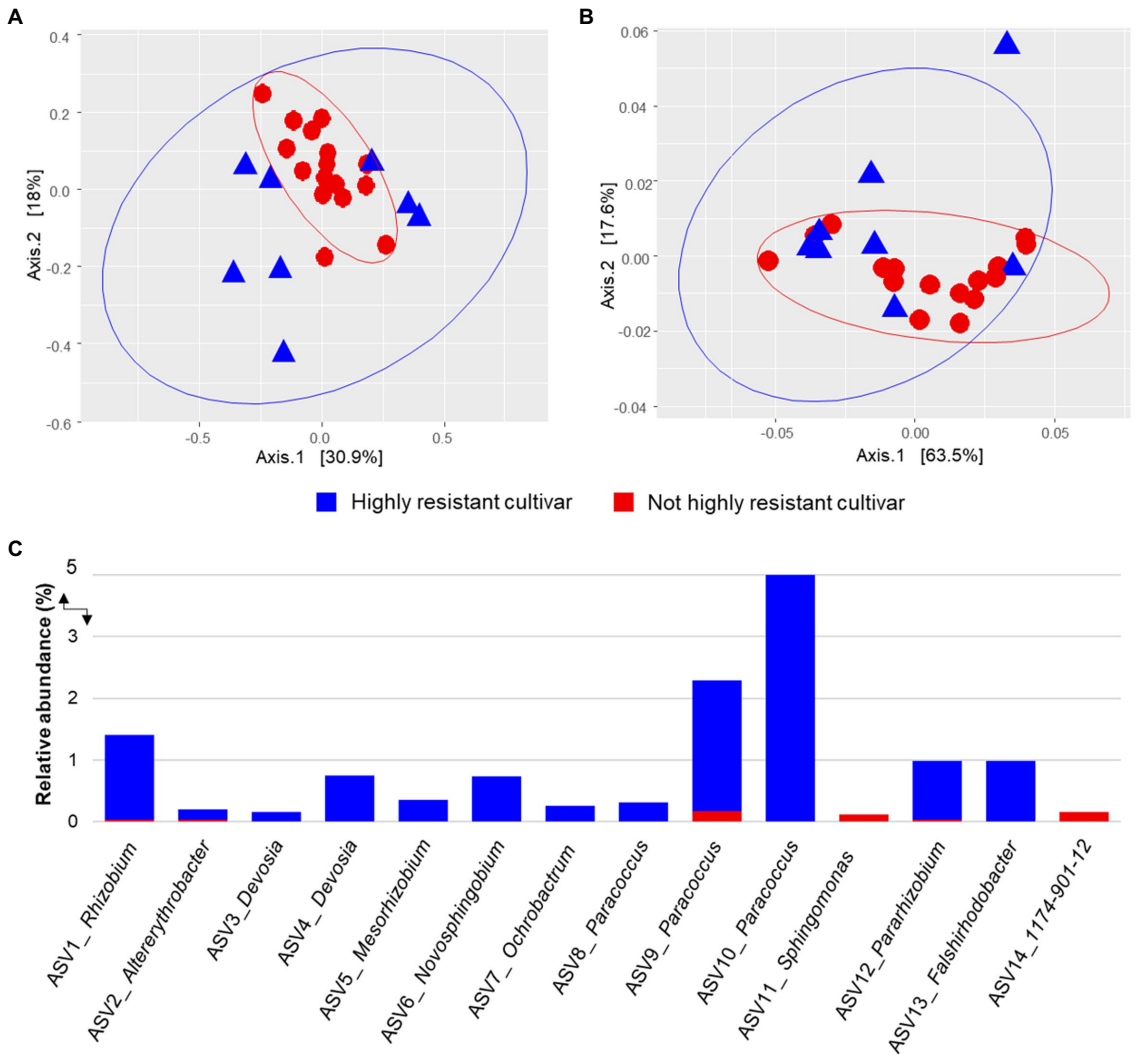
Alphaproteobacterial community structure of grapevine cultivars with different resistance (highly resistant, i.e., *V. amurensis* Rupr and *V. riparia* L. *vs* not highly resistant, i.e., *Vitis vinifera* L. subsp. *Sylvestris*, *Vitis vinifera* L. subsp. *vinifera “*Blauer Wildbacher,” *Vitis* × *alexanderi* Prince ex Jacques “Isabella,” and *Vitis vinifera* L. subsp. *vinifera* “Müller Thurgau”) traits against *Plasmopara viticola*. Alphaproteobacterial community clustering was assessed based on Bray–Curtis **(A)** and weighted UniFrac **(B)** matrix distances and visualized PCoA plots. Alphaproteobacterial ASVs that were differentially abundant between highly resistant cultivars (blue bars) and not highly resistant cultivar samples (red bars) according to edgeR analysis **(C)**.

Alphaproteobacterial community structures differed significantly between cultivars with high resistance toward *P. viticola,* i.e.*, V. amurensis* Rupr and *Vitis riparia* L and other cultivars (Bray–Curtis–ANOSIM, *R* = 0.321, *p* = 0.004; weighted UniFrac–ANOSIM, *R* = 0.25,7, *p* = 0.011) at harvesting period. Interestingly, at the flowering period, significant differences in alphaproteobacterial community structures between phenotype resistance toward *P. viticola* were not observed (Bray–Curtis–ANOSIM, *R* = 0.130, *p* = 0.150; weighted UniFrac–ANOSIM, *R* = 0.072, *p* = 0.254). PcoA plots were constructed using data from the harvesting period where the first two PCs explained 48.9 and 81.1% of the cumulative variances. The PcoA plot suggested two major clusters where the alphaproteobacterial community from the highly resistant cultivar was more scattered in comparison to the other cultivars that tend to group together (Figures 3A,B).

Due to differences in alphaproteobacterial community structures between grapevine cultivars with and without phenotype resistance toward *P. viticola* at the harvesting period, differential abundance analysis at the ASV level was performed. In total, 14 alphaproteobacterial ASVs were considered as biomarkers for distinguishing between the highly resistant cultivars and not highly resistant cultivars based on edgeR (Figure 2C). Twelve ASVs were enriched in highly resistant cultivar samples in comparison to not highly resistant cultivar samples. Of ASVs that were enriched in high resistance cultivars, they were identified as *Paracoccus* (*n* = 3 ASVs), *Devosia* (*n* = 2 ASVs), and *Allorhizobium-Neorhizobium-Pararhizobium-Rhizobium* (*n* = 2 ASVs). Two ASVs that were identified as *Sphingomonas* and *1,174–901-12 were enriched in* not highly resistant cultivar samples.

**Figure 4:**
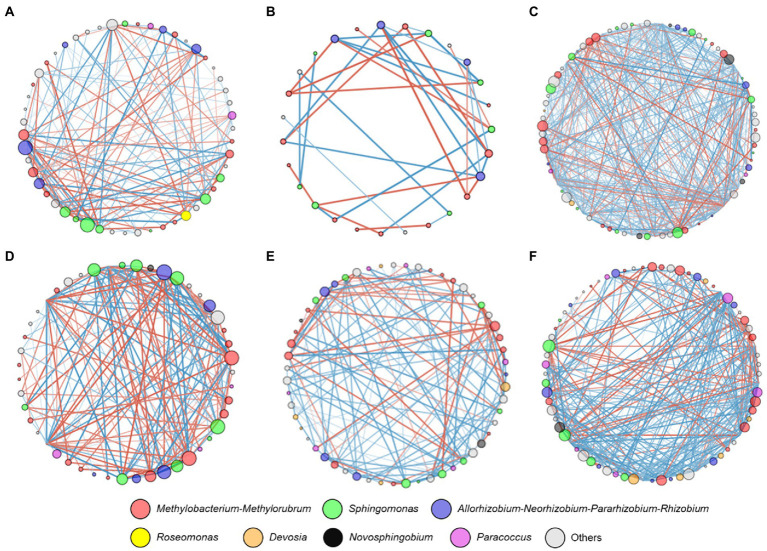
Network analyses of alphaproteobacterial communities. A connection stands for a significant (*p* < 0.05) correlation between two ASVs. Node sizes represent the number of connections (i.e., degree) and the thickness of lines connecting nodes represents the value of the Spearman correlation coefficients. Red lines represent negative correlations and blue lines represent positive correlations. Different colors represent different bacterial genera. **(A)** *Vitis vinifera* L. subsp. vinifera “Müller Thurgau;” **(B)** *Vitis x alexanderi* Prince ex Jacques “Isabella;” **(C)** *Vitis labrusca* L.; **(D)** *Vitis vinifera* L. subsp. *sylvestris* (C.C. Gmelin) Beger; **(E)** *Vitis riparia* L.; **(F)** *Vitis amurensis* Rupr.

Network analysis was conducted to further investigate the interrelationships between the bacterial taxa from different grapevine cultivars. The most connected nodes for bacterial taxa regardless of grapevine cultivars belonged to bacterial genera *Allorhizobium-Neorhizobium-Pararhizobium-Rhizobium*, *Methylobacterium-Methylorubrum,* and *Sphingomonas* (Figure 4). Interestingly, bacterial ASVs that belonged to *Devosia* showed positive correlations in highly resistant cultivars. These ASVs were also undetectable in other cultivars. According to topological parameters, a relatively higher network complexity, as indicated by a higher clustering coefficient, was observed from highly resistant cultivars (Clustering coefficient = 3.03 and 3.61, Table 2) in comparison to lowly resistant and susceptible cultivars (Clustering coefficient < 0.270). Highly resistant cultivars also had a higher ratio of positive–negative correlations (P/N = 2.4 and 3.2, Figures 4E,F) in comparison to other cultivars (P/N < 1.5), except, *V. labrusca* L (*p* = 3.6, Figure 4C). Moreover, a lowly resistant cultivar namely *V. labrusca* L presented the highest node connectivity (5.10) and was followed by a highly resistant cultivar namely *V. amurensis* Rupr. (4.08).

**Table 2:** Network topological properties of different grapevine cultivars.

Cultivar ID	*P. viticola* resistance level	No. of Node	No. of Edge		P/N^#^	Average degree^$^	Clustering coefficient
			Positive	Negative			
MT	Susceptible	51	82	62	1.3	2.82	0.204
AL	Lowly resistant	27	22	15	1.5	1.37	0.232
LA	Lowly resistant	87	347	97	3.6	5.10	0.270
SY	Moderately resistant	47	88	86	1.0	3.70	0.319
RI	Highly resistant	55	100	41	2.4	2.56	0.361
AM	Highly resistant	72	224	70	3.2	4.08	0.303

# P/N represents a ratio between positive and negative correlations.

$ Average degree represents the average number of edges per node in the graph.

MT, Vitis vinifera L. subsp. vinifera “Müller Thurgau;” AL, Vitis x alexanderi Prince ex Jacques “Isabella;” LA, Vitis labrusca L.; SY, Vitis vinifera L. subsp. sylvestris (C.C. Gmelin) Beger; RI, Vitis riparia L.; AM, Vitis amurensis Rupr.

## Discussion

In this study, we provide a deep insight into the microbial communities of the grapevine phyllosphere. We demonstrated that alphaproteobacterial richness and community composition were driven mainly by plant genotype and development stage. Interestingly, we identified a significant correlation between the composition of *Alphaproteobacteria* on mature grapevine leaves and the resistance level to *P. viticola*, one of the most devastating leaf pathogens in viticulture. In a detailed data assessment, we identified putative bacterial biomarkers, i.e., *Parococcus*, *Devosia,* and *Rhizobium* as potential bacterial biomarkers that are associated with the resistant cultivars.

We observed the native colonization patterns of *Rhizobiales* (*Alphaproteobacteria*) in the grapevine phyllosphere by FISH-CLSM particularly on young leaves with freshly emerged stomata that serve as bacterial hotspots. *Vitis labrusca* and the hybrid *V. alexanderi* “Isabella” (*V. labrusca* x *V. vinifera*) investigated in this study were covered densely with fine hair impairing the adherence of water on the leaf surface (Kortekamp et al., 1999), and likely affecting microbial colonization. In contrast, the susceptible cultivar “Müller Thurgau” has an extremely wettable leaf surface (Kortekamp et al., 1999). Bearing an inner cuticular rim within their stomata, *V. riparia* and *V. amurensis* differ morphologically from the other genotypes, which might also play a role in providing distinct niches for bacterial colonization.

Our findings indicate that the plant development stage was the main factor that affected alphaproteobacterial diversity and community structures (Figure 2). Here, we showed that grapevine leaves from the second sampling, conducted at the end of flowering in the consecutive spring, were generally characterized by higher richness and diversity of species. Previous studies have shown a shift in bacterial richness and community structure during plant development stages. For instance, bacterial richness in leaves of *Lactuca sativa* was higher after planting than at harvest (Dees et al., 2015). Moreover, bacterial community structures in leaves of *Leptospermum scoparium* were more uniform between mature plants in comparison to immature plants (Wicaksono et al., 2016). It should be noted that environmental factors and biogeographic variation can widely be excluded to act as drivers for shaping the “microbial wine terroir” (Verginer et al., 2010; Bokulich et al., 2014; Abdelfattah et al., 2019) in our study, as all plants (with the exemption of greenhouse-grown ‘Müller Thurgau’) were grown in close vicinity to each other and were exposed to similar microbial sources. We suggested that as leaves become mature, host plants only select a specific subset of bacterial taxa from the early developmental stage. Consequently, a reduction in bacterial richness and diversity was observed at the harvesting period. Moreover, grapevine cultivars also influence the alphaproteobacterial community structures and their effect was significant at the flowering period. Different alphaproteobacterial community structures between grapevine cultivars are presumably connected with niches formed by leaf morphological differences providing distinct microbial habitats as observed by FISH/CLSM. Our study provided evidence of a significant correlation between leaf morphology and microbiome assembly in particular alphaproteobacterial community composition.

The structure of leaf microbiome community at harvesting period was associated with phenotype resistance toward *P. viticola* (Figure 3). ASVs belonging to *Paracoccus*, *Devosia,* and *Allorhizobium-Neorhizobium-Pararhizobium-Rhizobium* were identified as microbial biomarkers for distinguishing between the highly resistant cultivars and not highly resistant cultivars. *Paracoccus* was found to be negatively correlated with downy mildew disease severity caused by *P. viticola* (Perazzolli et al., 2014) indicating possible biocontrol properties of some strains against downy mildew. Moreover, *Devosia* and *Rhizobium* were shown to have plant growth promotion traits (Sahoo et al., 2019; Deyett and Rolshausen, 2020; Paolinelli et al., 2022). Therefore, an increase in the relative abundance of these bacterial taxa in highly resistant cultivars may possess key functions for integrated crop protection. This information could be used to target and isolate specific taxa that can be used as potential biological control strains against *P. viticola*. Interestingly, resistant cultivars had a higher network complexity and a more positive correlation between bacterial taxa (Figure 4). Higher network complexity and positive correlation between bacterial taxa indicate higher synergism and stability of the bacterial communities (Zhang et al., 2018; Wassermann et al., 2019). Thus, it is speculated that higher network complexity and more positive correlations between bacterial taxa in resistant cultivars contribute to suppressing potential diseases outbreak in the grapevine phyllosphere.

In conclusion, our results align with the hypothesis that microbes contribute to the disease-resistance phenotype of their host plants which, in turn, provides the habitat for both pathogenic and beneficial microorganisms. Microbiome and co-occurrence studies focusing on crop plants are an important tool for the selection of biocontrol organisms and helper strains involved in microbe-microbe interactions. Core taxa correlated with host performance including plant health as well as host factors facilitating their long-term establishment and proliferation throughout the vegetative season represent promising targets for further investigations.

## Data availability statement

The data presented in the study are deposited in the European Nucleotide Archive (ENA) repository, accession number PRJEB59055.

## Author contributions

GB designed the study. CM and CD performed the field experiments and prepared the samples for sequencing. CM and WW analyzed the data. CM, WW, and GB wrote the manuscript. AA and HM critically reviewed the manuscript. WW and CM contributed equally. All authors contributed to the article and approved the submitted version.

## Funding

This work was partially funded by the Austrian Centre of Industrial Biotechnology (ACIB GmbH), which has been supported by the Austrian BMWFJ, BMVIT, SFG, Standortagentur Tirol, and ZIT through the Austrian FFG-COMET-Funding Program. Open access funding was provided by the TU Graz Open Access Publishing Fund.

## Conflict of interest

CM was employed by SAN Agrow Holding GmbH. CD was employed by Bio-Ferm GmbH.

The remaining authors declare that the research was conducted in the absence of any commercial or financial relationships that could be construed as a potential conflict of interest.

## Publisher’s note

All claims expressed in this article are solely those of the authors and do not necessarily represent those of their affiliated organizations, or those of the publisher, the editors and the reviewers. Any product that may be evaluated in this article, or claim that may be made by its manufacturer, is not guaranteed or endorsed by the publisher.
