# Supplementary material for: The mature phyllosphere microbiome of grapevine is associated with resistance against Plasmopara viticola
Source: Front Microbiol. 2023 Apr 11;14:1149307. doi: 10.3389/fmicb.2023.1149307 (PMC10127535; doi:10.3389/fmicb.2023.1149307)
Supplement: Supplementary file 1 [file Data_Sheet_1.docx]

Supplementary Material

**The mature phyllosphere microbiome of grapevine is associated with resistance against *Plasmopara viticola***

**Wisnu Adi Wicaksono^1*#^, Christina Morauf^2#^, Henry Müller^1,3,^, Ahmed Abdelfattah^1,3^, Christina Donat^4^, Gabriele Berg^1,3,5,6*^**

^#^both authors contributed equally

*** Correspondence:**Wisnu Adi Wicaksono and Gabriele Berg
E-mail: wisnu.wicaksono@tugraz.at; gabriele.berg@tugraz.at

**Supplementary Figure**

**Supplementary Figure S1 Rarefaction curves for alphaproteobacterial 16S rRNA gene amplicon libraries**
